# Understanding Epicardial Cell Heterogeneity during Cardiogenesis and Heart Regeneration

**DOI:** 10.3390/jcdd10090376

**Published:** 2023-09-01

**Authors:** Cristina Sanchez-Fernandez, Lara Rodriguez-Outeiriño, Lidia Matias-Valiente, Felicitas Ramírez de Acuña, Diego Franco, Amelia Eva Aránega

**Affiliations:** 1 Cardiovascular Development Group, Department of Experimental Biology, Faculty of Experimental Sciences, University of Jaén, 23071 Jaén, Spain; csfernan@ujaen.es (C.S.-F.); lrodrigu@ujaen.es (L.R.-O.); lmmatias@ujaen.es (L.M.-V.); fracuna@ujaen.es (F.R.d.A.); dfranco@ujaen.es (D.F.); 2 Medina Foundation, Technology Park of Health Sciences, 18016 Granada, Spain

**Keywords:** epicardium, heterogeneity, cardiac development, cardiac repair

## Abstract

The outermost layer of the heart, the epicardium, is an essential cell population that contributes, through epithelial-to-mesenchymal transition (EMT), to the formation of different cell types and provides paracrine signals to the developing heart. Despite its quiescent state during adulthood, the adult epicardium reactivates and recapitulates many aspects of embryonic cardiogenesis in response to cardiac injury, thereby supporting cardiac tissue remodeling. Thus, the epicardium has been considered a crucial source of cell progenitors that offers an important contribution to cardiac development and injured hearts. Although several studies have provided evidence regarding cell fate determination in the epicardium, to date, it is unclear whether epicardium-derived cells (EPDCs) come from specific, and predetermined, epicardial cell subpopulations or if they are derived from a common progenitor. In recent years, different approaches have been used to study cell heterogeneity within the epicardial layer using different experimental models. However, the data generated are still insufficient with respect to revealing the complexity of this epithelial layer. In this review, we summarize the previous works documenting the cellular composition, molecular signatures, and diversity within the developing and adult epicardium.

## 1. Introduction

The epicardium is an epithelial layer covering the surface of the heart that plays a key role in the prenatal development of the heart, constituting a crucial source of cells and signaling. During cardiac development, epicardial cells undergo epithelial-to-mesenchymal transition (EMT), giving rise to epicardium-derived cells (EPDCs) that are internalized into the subepicardial, myocardial, and subendocardial areas and differentiate into distinct types of cardiac cell, such as coronary vascular smooth muscle cells (vSMCs), cardiac fibroblasts (CFs), or endothelial cells (ECs) [1,2,3,4]. Although the epicardium remains a quiescent layer during adulthood, it can be reactivated in response to cardiac injury via the upregulation of its embryonic developmental genetic program [5,6]. Thus, the epicardium constitutes an intriguing cell population of multipotent progenitors to study, and it may contribute to cardiac wound healing via regulating tissue remodeling after heart damage.

However, despite its essential role during embryonic development, in the maintenance of cardiac homeostasis, and after cardiac injury, fundamental insights into the epicardium’s cell heterogeneity and functional crosstalk with other cell types are currently lacking. Furthermore, little is known about how different cell types emerge from EPDCs or the molecular mechanisms underlying EPDCs’ cell fate during the development and reactivation after heart damage. In recent years, different approaches have been used to study cell heterogeneity and molecular signatures within the epicardial cell layer in embryonic and adult hearts. Despite this knowledge, there are many unanswered questions regarding epicardial cell behavior, and a better understanding of the role of this epithelial layer during development and with respect to heart diseases is necessary. Herein, we provide a state-of-the-art review of current studies on epicardial cell heterogeneity as assessed in different experimental models during embryonic development and adulthood.

## 2. Epicardium during Cardiac Development and Regeneration

### 2.1. Epicardial Contribution to Heart Development

An epithelial layer of cells covering the pericardial surface of the heart can be observed in the vertebrate heart. This evolutionarily conserved structure, called the epicardium, mainly originates from the proepicardium (PE) and harbors a population of progenitor cells that undergo EMT prior to differentiating into distinct cardiac lineages [7,8,9]. During this transition, epithelial cells lose their cell–cell adhesion and their apical–basal polarity, acquiring migratory and invasive characteristics of mesenchymal stem cells that allow for their internalization [9,10,11]. Once epicardial cells have been internalized, the delaminated EPDCs differentiate into specialized cells, including coronary vSMCs, CFs, ECs, and, presumably, a subpopulation of CMs [2,3,4,12]. 

However, the role of the epicardium during cardiac development is not limited to serving as a progenitor source contributing to multipotent cells that give rise to cardiac mesenchyme. This epithelial layer is also a source of paracrine cues and extracellular matrix (ECM) components that are essential for fetal cardiac growth or coronary vessel patterning [13]. In this context, epicardium-derived FGF (Fibroblast growth factor) signals and the production of chemokines such as CXCL12 (*C-X-C motif chemokine 12*) can regulate myocardial proliferation and coronary vessel maturation, respectively [14,15]. Thus, the epicardium and myocardium exchange bidirectional signals, which are crucial for the normal growth of the heart muscle and the development of coronary vessels [16]. 

After birth, the postnatal epicardium enters a deceptively quiescent state, serving as a barrier between the myocardium and the pericardial cavity. 

### 2.2. Epicardial Response to Damage: Cardiac Repair

In response to cardiac damage, the adult epicardium reactivates an embryonic-like response in which developmental gene programs, including *Wt1* (*Wilms’ tumor 1*) and *Tbx18* (*T-box transcription factor 18*), are upregulated to modulate tissue repair [6,16,17,18]. This potential contribution of adult epicardial cells to cardiac repair was initially observed in zebrafish, where a resection of the apex of the heart led to the activation of epicardial cells and the subsequent EMT of EPDCs [18]. The migration of these cells into the damaged tissue allows for their differentiation into vascular cells to support the regeneration of the injured area [19]. Additionally, *Wt1a* and *Wt1b* lineage reporter models have further revealed that the epicardium also contributes to cardiac fibroblast plasticity during zebrafish heart regeneration [20].

In murine models, the acute upregulation of embryonic and EMT marker genes is detected in adult reactivated epicardia in response to myocardial injury [6,16,17,18]. However, unlike what happens in the embryonic context, adult EPDCs have a limited regenerative capacity and preferentially differentiate into cardiac fibroblasts and smooth muscle cells [16,21,22]. Furthermore, in recent years, the use of lineage-tracing models has revealed that most of the newly differentiated cells that appear after cardiac injury arise from preexisting cardiac cell subpopulations, such as SMCs, ECs, or CMs [23,24,25,26,27]. These results suggest that the post-injury contribution of EPDCs appears to be less efficient when compared to their role during development [17,28]. Thus, the adult epicardium is thought to serve more as a source of paracrine signals rather than a source of “reparative” cells [17,29,30]. For instance, in injured adult zebrafish hearts, activated *Igf2b* (*Insulin growth factor 2b*) is observed on the epicardial surface and in surrounding apical wounds, suggesting that IGF paracrine signaling is required for the proliferation of CMs during wound repair [30,31]. Similarly, follistatin-1 produced by epicardial cells increased cell cycle re-entry and the division of pre-existing cardiomyocytes in mouse and swine models of myocardial infarction [32]. 

In addition to the information described above, it should not be forgotten that the epicardium is a crucial agent in relation to cardiac development and tissue remodeling after heart damage. Although the epicardium has been proposed as a potential target in the treatment of cardiovascular disease in recent years, a deeper understanding is needed to unlock its full potential as a source of cells and signals that satisfactorily complete the reparative process.

## 3. Determining Epicardial Heterogeneity: Do Different Cell Types Constitute the Epicardial Layer?

The identification of epicardial cell heterogeneity and cellular intercommunication can play a vital role in differentiating healthy hearts from diseased hearts, and it may predict future outcomes with superior precision and at earlier stages of cardiac failure. Fluorescence-activated cell sorting (FACS) is a powerful tool that allows for the identification of specific cells; however, it either has limitations in terms of sample preparation or a paucity of markers that can be assayed simultaneously [33]. Therefore, FACS is not suitable for the subclassification of unknown cells and the identification of their potential functions. Although Cre-based lineage-tracing models have recently been used to understand the cell fate of EPDCs, most of them are not sufficiently specific to the epicardial layer and have been supplemented or replaced by single-cell RNA-sequencing (scRNA-seq) techniques [5]. These methods have enabled us to gain a much more detailed view of novel individual cellular phenotypes, identifying new cell types and subpopulations with distinct cellular expression patterns by integrating the transcripts of different genes, even within an apparently homogeneous cell population [34,35]. In this context, by using scRNA-seq and transcriptomic analyses, Weinberger et al. (2020) have described three epicardial cell subpopulations with distinctive spatial distributions during cardiac development in zebrafish embryos; these phenomena were observed concretely on day 5 post-fertilization [36] (Table 1). Interestingly, only one of these transcriptionally distinct epicardial cell subpopulations contained cells co-expressing the prototypical epicardial signature genes *Tcf21* (*Transcription factor 21*), *Tbx18*, and *Wt1b.* In addition, this cell cluster expressed high levels of *Tgm2b* (*Transglutaminase 2b*), which serves to ensure cell–cell contact and plays a critical role in maintaining the integrity of the developing epicardium. The second subpopulation was enriched with genes associated with the regulation of epicardial cell contribution to the smooth muscle layer of the outflow tract such as *Tbx18*, *Acta2* (*Actin alpha 2*), and *Mylka* (*Myosin light chain kinase a*). These scRNA-seq analyses defined a third cell cluster spatially restricted to an area between the bulbus arteriosus (BA) and the atrium that was characterized by a high expression of genes involved in leukocyte chemotaxis and guidance cues [36]. Altogether, the appearance of three distinct cell subpopulations, with different localizations and functions, suggests that the epicardium in the developing zebrafish heart is a heterogenous cell layer. 

In developing mammals, such as mice and humans, epicardial cells heterogeneously express the transcription factors *Tcf21* and *Wt1*. Both are also expressed in PE cells and downregulated in the adult epicardium but reactivated after myocardial ischemic injury [37,38]. However, little is known about the molecular regulation of epicardial cell heterogeneity and its different functions. In this context, scRNA-seq assays of EPDCs derived from human pluripotent stem cells have identified basonuclin (BNC1) as an upstream regulator of a transcriptional hierarchy regulating cell identity in the developing epicardium. This transcription factor is expressed in the adult epicardium and downregulated after myocardial injury. BNC1 has been identified, together with the membrane protein THY1 (*Thy-1 cell surface antigen*), as a marker of different epicardial subpopulations [37,39]. Thus, TCF21^high^/THY1^+^ cell populations had a higher propensity to become CFs, whereas BNC1^high^ cell clusters expressed genes involved in muscle differentiation, migration, and cell–cell interaction [37] (Table 1).

As mentioned above, scRNA-seq is a powerful tool for studying the cellular mechanisms involved in heart development, allowing us to characterize different cell types and generating hypotheses about their origins and fates [40]. Therefore, scRNA-seq enables the dissection of cellular heterogeneity in an unbiased manner, with no need for any prior knowledge of a cell population [35]. However, this recent approach does not preserve spatial information about tissue morphology and cellular interactions [40]. Thus, to obtain more detailed analyses that can provide us with a more global view of epicardial development, scRNA-seq studies have been combined with spatiotemporal transcriptomics, in which several time points within a studied period have been considered. In this context, two heterogeneous epicardial cell subpopulations expressing genes that encode integral membrane proteins such as *Upk1b* (*Uroplakin-1b*) and *Upk3b* (*Uroplakin-3b*) have been identified in the outflow tract region between the E11.5 and E13 stages in mice [41,42] (Table 1). In a similar study integrating scRNA-seq and spatial RNA-seq data, epicardial cells were identified in five main cell clusters based on their positions within different Hamburger–Hamilton ventricular development stages in a chicken embryo [43] (Table 1). The time points analyzed represented key steps of epicardial development ranging from the state of the epicardium prior to EMT (HH24), during EMT (HH31), and during/after epicardial differentiation (HH36 and HH40). Thus, at HH24, an early epicardial progenitor cell cluster, characterized by the expression of canonical epicardial progenitor markers such as *Tcf21*, *Tbx18*, and *Wt1*, was identified as being restricted to the epicardial layer of the ventricular walls. In this region, another subpopulation enriched in *Bmp4* (*Bone morphogenetic protein 4*) and *Lum* (*Lumican*) was described at HH31, during the EMT. In the third cell cluster described within the myocardium, high levels of extracellular matrix transcripts involved in cell migration were observed at the same stage. Interestingly, as Mantri et al. (2021) have suggested, this subpopulation may represent a mesenchymal phenotype of EPDCs that are undergoing EMT and migrating into the myocardium. At HH36, a fibroblast-like cell cluster, which expressed *Col3a1* (*Collagen type III*), and a mural cell cluster enriched, among others, in *Acta2* and *Myh11* (*Myosin heavy chain 11*) were present within the myocardium. Furthermore, at the same stage, some cells of the outermost epicardial layer still maintained an undifferentiated phenotype [43].

Although no functional epicardial cell heterogeneity was observed in the study by Mantri et al. (2021), a variation in the differentiation state was described, as cells may continue to stay in the epicardium with an undifferentiated intermediate phenotype during later stages of development, at which point EMTs will have been initiated. Thus, their data led them to hypothesize that EPDCs that undergo EMT maintain a progenitor-like transcriptional profile before their fate specification in the myocardium [43]. These results suggest that the observed heterogeneity does not stem from differences in the initial cell population. Although what triggers EPDCs to undergo EMT at specific developmental times and locations remains unknown, this study suggests that ECM cues are significantly involved in this process [43]. Similarly, recent studies have demonstrated that ECM is a key player in EMT and EPDC generation in the developing mouse heart. Interestingly, recent scRNA-seq analysis of a developing murine heart supports the notion that epicardium-derived cell fate, namely, the formation of fibroblasts or vSMCs, is specified by *Tcf21* expression and PDGF-B endothelium-mediated signaling, respectively [44]. These data suggest that epicardium-derived cell fate is specified only after EMT, seemingly in response to environmental cues, and that, importantly, marker expression profiles do not restrict cell fate choice [44].

Although cellular heterogeneity is a general feature of biological tissues that has been identified through sequencing analyses of multiple organisms, even within an apparently homogeneous cell population, some controversial results have been observed linked to cellular heterogeneity within the epicardial layer [35]. Thus, the conversion of early epicardial Wt1^high^/Tcf21^high^ cells to Wt1^low^/Tcf21^high^ mesenchymal cells during EMT reflects a developmental transition rather than the heterogeneity of the starting population in the developing murine heart. These results suggest that the different subpopulations that have been described are more likely a result of developmental progression and that the fate of EPDCs is specified after EMT, potentially in response to extrinsic cues like paracrine factors or ECM [44]. In contrast, similar analyses of the human heart at different time points during development (4.5–5-, 6.5-, and 9-weeks post-conception) showed that the epicardium displays less gene expression heterogeneity compared to more undifferentiated mesenchymal cells (Table 1). However, the authors argued that the low number of sequenced cells and the limited number of genes analyzed were insufficient for a detailed comprehension of what occurs in epicardial cells throughout the period under study [45].

**Table 1:** Subtypes of epicardial cells identified, during development, in different experimental models.

Cell Population	Markers	Organism	Function	Reference
Epi1	*Tcf21*, *Tbx18*, *Wt1b*, *Tgm2b*	Zebrafish (5-dpf)	Maintain epicardial integrity	[36]
Epi2	*Tbx18*, *Acta2*, *Mylka*, *Sema3fb*		Contribution to smooth muscle layer	
Epi3	*Tcf21*, *Cxcl12a*		Leukocyte chemotaxis	
BNC1^+^/TCF21^low^	*Bnc1*, *Tcf21*, *Wt1*	Human	Muscle differentiation, migration, and cell–cell interaction	[37]
THY1^+^/TCF21^high^	*Thy1*, *Tcf21*		Potential to become cardiac fibroblasts	
C10	*EP genes*, *Myl7*, *Tmem255a*	Mouse (E11.5–13)		[41]
C11	*EP genes*, *Ein*, *Dlk1*, *Klk14*			
C1	*Tcf21*, *Tbx18*, *Wt1*	Chicken (HH24–40)		[43]
C2	*Tcf21*, *Tbx18*, *Wt1*, *Mdk*, *Bmp4*, *Lum*			
C3	*Tcf21*, *Fn1*, *Postn*, *Egfl7*, *Agrn*, *Snai1*			
C4	*Tcf21*, *Col3a1*, *Dcn*			
C5	*Acta2*, *Myh11*, *Tagln*, *Rgs5*			
C3	*Tbx18*, *Tcf21*	Human (4.5–9 pcw)	EPDCs	[46]
C9	*Aldh1a2*, *Lrp2*, *Itln1*, *Tbx18*		Epicardial cells	

EP = epicardial, dpf = days post-fertilization, and pcw = post-conception weeks. *Myl7* (*Myosin light chain 7*), *Tmem255a* (*Transmembrane protein 255A*), *Ein* (*Elongated internode*), *Dlk1* (*Delta like non-canonical notch ligand 1*), *Klk14* (*Kallikrein-related peptidase 14*), *Mdk* (*Midkine*), *Fn1* (*Fibronectin-1*), *Postn* (*Periostin*), *Egfl7* (*EGF-like domain multiple 7*), *Agrn* (*Agrin*), *Snai1* (*Snail family transcriptional repressor 1*), *Dcn* (*Decorin*), *Tagln* (*Transgelin*), *Rgs5* (*Regulator of G-protein signaling 5*), *Aldh1a2* (*Aldehyde dehydrogenase 1*), *Lrp2* (*LDL-receptor-related protein 2*), and *Itln1* (*Intelectin 1*).

In contrast to the developing epicardium, in the postnatal period, this epithelial layer maintains a quiescent state characterized by a reduced expression of developmental genes such as *Wt1* and *Tbx18*. Furthermore, no cellular contribution to the myocardium has been observed in this quiescent state [16,18,46]. Nevertheless, in response to cardiac damage, the epicardium suffers a reactivation initiating an embryonic-like response [16,18]. Thus, reactivated epicardial cells can form a multi-cell layer of epicardial stromal cells (EpiSc) on the surface of the heart that secretes paracrine factors to stimulate cardiomyocyte growth, angiogenesis, and adaptive immune regulation [16,18,47]. In this context, FGF2 and VEGFA (*Vascular endothelial growth factor A*) secreted by EPDCs have been described to increase vessel density after myocardial infarction in a mouse model [16]. Hence, the epicardium plays a crucial role as a signaling center, serving as a resident population of progenitor cells involved in cardiac repair, carrying out cell repopulation of the damaged area, and promoting regenerative responses against cardiac injury [46,48]. For instance, in the Wt1^CreERT2/+^; ROSA26-tdT^+/−^ murine model of atrial cardiomyopathy, reactivated epicardial cells differentiate into myofibroblasts during tissue remodeling [49].

Although it is generally assumed that the adult activated epicardium recapitulates an embryonic-like response characterized by the generation of mesenchymal progenitor cells, after ischemic insults or cardiac injury, there may be important differences concerning their embryonic phenotypes [50]. In the adult heart, the reparative process appears to be less efficient with regard to EPDC migration and differentiation, producing signals that activate the proliferation of resident CFs and fibrosis induction [51]. Furthermore, it remains unknown whether this disparity in regenerative capacity could derive from differences in the cellular composition of the epicardium due to the presence of subsets of cells that contribute to cardiac repair to a greater extent. In this context, several groups have made significant efforts to better understand cell heterogeneity and molecular signatures within the epicardial layer of the adult heart. For instance, Cao et al. (2016) have reported three different cell subsets defined by distinct gene expression signatures in a *Tcf21*^+^ epicardial population isolated from adult zebrafish ventricles during regeneration (Table 2). One subpopulation represents the outermost epithelial epicardial cells; the second cell cluster, composed of internal cells ubicated in the middle of the ventricular wall, was characterized by likely containing perivascular components; and the third one formed an innermost layer of EPDCs [52].

In mammals, epicardium activation after MI induction and thymosin beta 4 (Tβ4) administration revealed the presence of distinct subpopulations of EPDCs in the activated epicardium with variated cardiovascular potentials and molecular phenotypes distinct from embryonic EPDCs (E12.5) [50,53] (Table 2). However, it is important to highlight that the origins of the different cell populations observed in these studies are unclear, and it remains to be elucidated whether such cell heterogeneity is already present within the “quiescent” epicardial layer. 

In recent years, scRNA-seq combined with RNA in situ hybridization and lineage tracing has allowed for the identification of 11 transcriptionally distinct EpiSC populations isolated from mouse hearts after MI, and these populations can be classified into three independent groups [54] (Table 2). Based on data pertaining to these populations, it has been hypothesized that they are involved in the secretion of paracrine factors and the attraction of monocytes and neutrophils during the modulation of the innate immune response post-MI and that they exhibit a fibroblast-like phenotype [54]. However, despite analyses using a high-resolution technique, these results did not assess the function of the identified cell subpopulations and came from a singular timepoint analysis. However, these results bear some resemblance to the functionally heterogeneous epicardial subpopulations described in zebrafish by Weinberger et al. [36,54].

**Table 2:** Subtypes of epicardial cells identified in different experimental models of cardiac injury.

Cell Population	Markers	Organism	Function/Role	Reference
*I*	*Tcf21*, *Raldh2*, *Bmp4*, *Tfa*	Zebrafish (4 and 12 months)	Epithelial epicardial cells	[52]
II	*Tcf21*, *Nrg1*, *Col18a1*, *Ltbp1*		Perivascular components	
III	*Tcf21*, *Cxcl12a*		EPDCs	
*Wt1^+^*, *Sca-1^+^*	*Wt1*, *Sca-1*, *Gata4*, *Flk1*, *Sma*, *Fap*α	Mouse (2,4 and 7d post-MI)	Myofibroblast potential	[50]
*Wt1^+^*, *Sca-1^+^*, *CD90^high^/CD44^high^*	*Wt1*, *Sca-1*, *Cd90*, *Cd44*, *Isl1*, *Gata4*, *Flk1*		Cell–cell interaction, cell adhesion or migration	
EpiSC-1	*Wt1*, *Sema3d*, *Aldh1a2*, *Gata5*, *Tbx18*, *Tgm2*, *Sema3f*, *Mesp1*, *Gata4*, *Hif1a*	Mouse (5d post-MI)	Secretion of paracrine factors	[55]
EpiSC-2	*Tbx18*, *Cxcl12*, *Top2a*, *Mesp1*, *Gata4*, *Hif1a*		Attraction of monocytes and neutrophils	
EpiSC-3	*Tcf21*, *Pcsk6*, *Mesp1*, *Gata4*, *Mef2c*, *Bmp2*, *Bmp4*		Potential of cardiac fibroblasts	
EpiSC-4	*Tbx18*, *Cd44*, *Mesp1*, *Gata4*, *Hif1a*		Attraction of monocytes and neutrophils	
EpiSC-5	*Tcf21*, *Mesp1*, *Gata4*, *Mef2c*, *Bmp2*, *Bmp4*		Potential of cardiac fibroblasts	
EpiSC-6	*Tcf21*, *Sfrp2*, *Pcsk6*, *Mesp1*, *Gata4*, *Mef2c*, *Bmp2*, *Bmp4*		Potential of cardiac fibroblasts	
EpiSC-7	*Wt1*, *Sema3d*, *Aldh1a2*, *Gata5*, *Tbx18*, *Tgm2*, *Sema3f*, *Msln*, *Mesp1*, *Gata4*, *Hif1a*		Secretion of paracrine factors	
EpiSC-8	*Wt1*, *Tbx18*, *Mylk*, *Tmsb4x*, *Smarca4*, *Mesp1*, *Gata4*, *Hoxa5*, *Hif1a*		Attraction of monocytes and neutrophils	
EpiSC-9	*Tbx18*, *Tcf21*, *Top2a*, *Mef2c*, *Bmp2*, *Bmp4*		Potential of cardiac fibroblasts	
EpiSC-10	*Dkk3*, *Mef2c*, *Bmp2*, *Bmp4*		Potential of cardiac fibroblasts	
EpiSC-11	*Tbx18*, *Ifit3*, *Hif1a*, *Ccl2*, *Ccl7*, *Cxcl10*		Attraction of monocytes and neutrophils	

*Raldh2* (*Retinaldehyde dehydrogenase 2*), *Tfa* (*Tail fiber assembly*), *Nrg1* (*Neuregulin 1*), *Col18a1* (*Collagen type XVIII alpha 1*), *Ltbp1* (*Latent-transforming growth factor beta-binding protein 1*), *Sca-1* (*Stem cell antigen-1*), *Gata4* (*GATA binding protein 4*), *Flk1* (*Flt-related receptor tyrosine kinase 1*), *Sma* (*Smooth muscle actin*), *Fapα* (*Fibroblast activation protein alpha*), *Cd90* (*Cluster of differentiation 90*), *Cd44* (*Cluster of differentiation 44*), *Isl1* (*Isl lim homeobox 1*), *Aldh1a2* (*Aldehyde dehydrogenase 1*), *Gata5* (*GATA binding protein 5*), *Tgm2* (*Transglutaminase 2*), *Sema3f* (*Semaforin 3F*), *Mesp1* (*Mesoderm posterior 1*), *Hif1a* (*Hypoxia inducible factor 1 subunit alpha*), *Top2a* (*DNA topoisomerase II alpha*), *Pcsk6* (*Proprotein convertase subtilisin/kexin type 6*), *Mef2c* (*Myocyte enhancer factor 2c*), *Bmp2* (*Bone morphogenetic protein 2*), *Bmp4* (*Bone morphogenetic protein 4*), *Sfrp2* (*Secreted frizzled related protein 2*), *Msln* (*Mesothelin*), *Tmsb4x* (*Thymosin beta 4 x-linked*), *Smarca4* (*SWI*/*SNF-related matrix-associated actin 4*), *Hoxa5* (*Homeobox A5*), *Dkk3* (*Dickkopf WNT signaling pathway inhibitor 3*, *Ifit3 (Interferon-induced protein with tetratricopeptide repeats 3*), *Ccl2* (*CC motif chemokine ligand 2*), and *Ccl7* (*CC motif chemokine ligand 7*).

## 4. Does the Embryonic Origin of the Epicardium Underlie Its Cell Heterogeneity?

As mentioned above, during development, a subset of epicardial cells undergoes an EMT and migrates into the subepicardial space to give rise to several cardiac cell types. The fact that epicardial cells in the developing heart can differentiate into distinct cell subpopulations suggests that this epithelial layer is not composed of only one specific cell type. The hypothesis of the presence of different cell clusters along the epicardial layer can be supported by the notion that multiple tissues contribute to this layer’s embryonic origin. In this context, it has been proposed that the observed epicardial heterogeneity may be attributed, at least in part, to the origin and cellular composition of the PE or proepicardial organ (PEO), a heterogeneous cell mass from which most epicardial cells originate, which could influence the multiple cell fates of EPDCs during heart development [55].

Therefore, the PE is a primitive extracardiac organ formed as an outgrowth of the coelomic mesothelium located between the heart and the liver [56]. In mice, on embryonic day 8.5 (E8.5), the PE is situated at the base of the venous inflow tract of the developing primitive heart and does not interact directly with the myocardium [57,58,59]. The PE has an outer layer of epithelial cells expressing well-characterized proepicardial markers such as *Wt1* or *Tbx18*. These cells, which originate from precursors of the early cardiac progenitor fields that express the transcription factors *Nkx2.5* (*Homeobox protein Nkx2.5*) and *Isl-1* (*Insulin gene enhancer protein Isl-1*), overlie an inner core of several mesenchymal cell types and ECs [60]. In the PE, these different molecular markers have a heterogeneous spatiotemporal expression, potentially indicating the existence of several subtypes of cells with distinct roles that divide the PE into genetically distinguishable cell sub-compartments. Therefore, apart from *Tbx18*- and *Wt1*-expressing cell populations, lineage-tracing experiments conducted on mice and chickens have shown that some proepicardial cells from the mesenchymal core partially express other markers such as Scx (*Scleraxis*) and *Sema3D* (*Semaphorin 3D*) [44,61]. Curiously, in early stages, the *Scx*^+^ cell population contributes to the formation of the endocardium, and *Sema3D*^+^ cells contribute to the formation of the endothelium of the sinus venosus. It is important to note that both tissues are linked to the formation of the endothelium of coronary vasculature at later stages [61].

**Figure 1:**
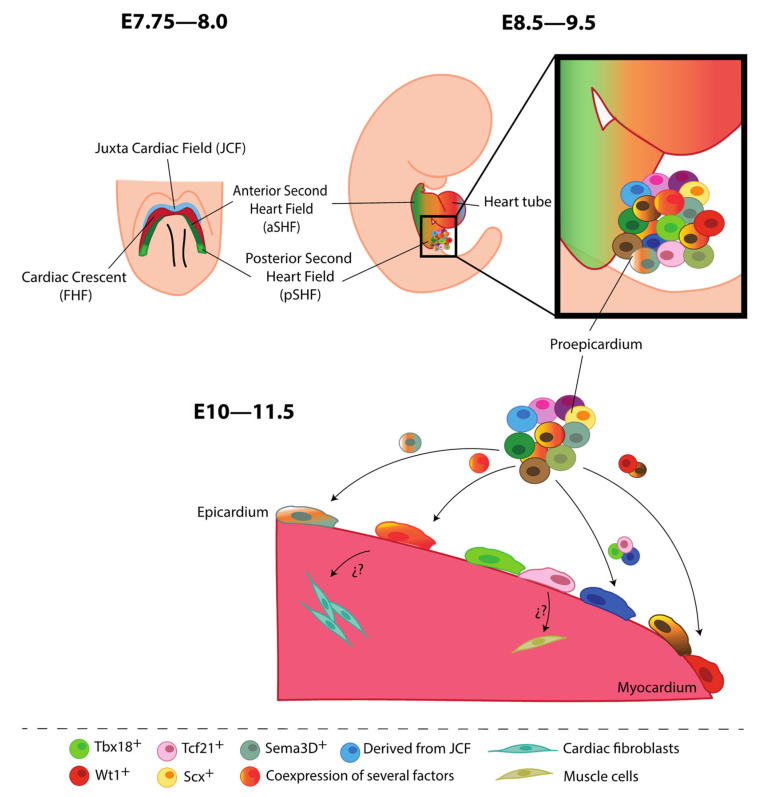
Cell heterogeneity of the epicardium during heart development. At E7.75–8.0, cardiac progenitor cells extend towards the midline to form the cardiac crescent or first heart field (FHF, shown in red) in the caudal direction with respect to the headfolds. In the rostral direction with respect to the FHF, there is a population of progenitor cells, constituting the juxta-cardiac field (JCF, shown in light blue). The second heart field (SHF) is composed of two subdomains: anterior (shown in dark green) and posterior (illustrated in light green). At E8.5–9.5, cardiac crescent fusion and posterior looping at the midline form the early cardiac tube. PE is situated in the venous inflow tract in the developing primitive heart. At around E10–11.5, clusters of PE cells begin to proliferate and spread, covering the heart tube with an epithelial layer, the epicardium. New experimental data have proposed (question marks) that EPDCs with specific cell fates (fibroblasts or muscle cells) might originate from different epicardial cell subpopulations.

In addition to the previously stated information, it has been shown that the PE is an important transient structure that contributes to different types of cells from various cardiac lineages; for instance, studies using avian and mammalian models have established that this compartmentalized structure is a source of vSMCs and CFs [61,62]. Other works assert that the PE also influences cardiomyocytes; however, this finding is subject to debate, as the lineage-tracing models used in these studies are not specific enough and may mislabel cells that do not necessarily originate from the PE [60,61,62]. In this context, Cossett and Misra (2011) have identified three different populations of endothelial cell precursors within the PE that appear to have distinct origins, such as the developing liver bud or the sinus venosus, suggesting that the PE “per se” may also act as a source of ECs [63]. Thus, there is previous evidence highlighting the possibility that the primitive PE organ might possess distinct cell sub-compartments containing different cardiac cell precursor populations that differ in both their routes and timing of migration to the heart to first constitute the epicardium and, later on, give rise to distinct, albeit overlapping, cell fates [61]. However, recent findings contrast with the idea of proepicardial sub-compartments reported by Katz et al. (2012), suggesting that, in mice, all PE and epicardial cells co-express *Wt1*, *Tbx18*, *Tcf21*, *Scx*, and *Sema3D* until embryonic stage E13.5 (Figure 1). Additionally, the minimal variation in the expression levels of these canonical markers between cells does not provide evidence for the existence of distinct areas in the PE or epicardial layer [44].

Once the embryonic heart has looped, clusters of PE cells begin to proliferate and spread, covering the bare heart tube with an epithelial layer, i.e., the epicardium [64]. It is important to highlight that the way PE cells migrate toward the heart differs between species. For instance, in chicks or frogs, proepicardial cells migrate via extracellular matrix bridges, while in mammals, cells from the PE form cell aggregates or vesicles that float through the pericardial cavity to colonize the myocardial surface [65,66,67,68]. When the epicardium completely covers the heart, namely, at around E11.5 in mice and at week 5 in human embryos, it constitutes a heterogeneous multicellular epithelium lining the ventricles, expressing different specific proteins such as WT1, TBX18, TCF21, GATA5 (*GATA binding protein 5*), and cytokeratin [60,62,69,70,71] (Figure 1). Furthermore, other clusters, such as CD45^+^ cells, have been identified within the epicardium. However, whether the different cell populations identified in the PE represent distinct stages of cell differentiation or constitute different cell precursors that give rise to specific cell populations remains to be investigated [72]. 

Although most epicardial cells originate from a heterogeneous cell mass called the PE, part of the epicardium covering the BA in zebrafish was found to be derived from the pericardial sac [73,74,75]. Furthermore, a non-PE-derived epicardial covering of the intrapericardial great arteries has been described in avian embryos [74,76,77]. In the developing mouse heart, Tyser et al. (2021) have described a novel source of proepicardial cells derived from the juxta-cardiac field (JCF), namely, a population of progenitor cells located rostrally with respect to the cardiac crescent, representing the earliest known progenitors of the epicardium. Using single-cell resolution time-lapse imaging and genetic lineage labeling, this group established that the JCF, characterized by *Mab21l2* (*Male-abnormal 21-like 2*) expression, constitutes a cardiac progenitor cell pool that is spatially and transcriptionally distinct from the sinus venosus progenitors. Thus, JCF progenitor cells may have specific abilities to differentiate into CMs within the linear heart tube, and they may also contribute to the PE. However, it is still unclear whether the JCF population is composed of unipotent progenitors of CMs and proepicardial cells or contains bipotent cells capable of giving rise to both cell types [40] (Figure 1). 

The data described above have highlighted some still unsolved questions about the complexity of the origin of epicardial heterogeneity. For example, is the colonization of myocardial surfaces by PE cells connected to epicardial heterogeneity? Could additional embryonic structures serve as new origins of proepicardial cells? To resolve these and other lingering questions, additional studies are required in order to shed light on the role of the PE in epicardial heterogeneity.

## 5. Reflections and Future Perspectives

Far from being just the outermost layer of the heart, the epicardium plays an essential role during cardiac development, serving as a source of cardiac cells and secretory and paracrine factors. As previously mentioned, during heart development, a subset of epicardial cells loses their apical–basal polarity and cell–cell adhesion when undergoing an EMT, which allows for the formation of EPDCs and their differentiation into various cell types such as fibroblasts or smooth muscle cells [2,3,10,11]. However, it remains unclear whether pre-migratory EPDCs are a homogeneous group of multipotent cell progenitors or if they are somehow specified as epicardial subpopulations within the epicardium before migration. In addition, many other questions remain unanswered regarding epicardial cell behavior during the EMT process. For instance, why do some epicardial cells undergo an EMT, while others remain as epithelial cells? What are the molecular mechanisms that drive epicardial cell dynamics during the process of EMT? 

After a cardiac injury, this epithelial layer, which remains in a quiescent state during adulthood, recapitulates embryonic capabilities, providing the epicardium with the ability to contribute to cardiac repair. Although contributing in a less efficient manner compared to its role during development, the epicardium has been considered a very interesting actor in endogenous cardiac remodeling after damage. However, it is still unclear whether specific epicardial cell subpopulations can participate in cardiac remodeling or whether distinct cell types residing within the epicardial layer have determined capabilities during the reparative response [5]. In this context, several studies conducted over the last few years have focused on understanding epicardial composition as well as the molecular mechanisms underlying epicardial cell fate decisions in order to identify targets to optimize the post-injury response in the adult heart. However, it is common for the potential heterogeneity of the epicardium to be overlooked due to a low number of epicardial cells, few developmental stages under study, or incorrect markers used to identify these novel subpopulations [78,79]. These factors, along with fluctuations in the abundance of diverse cellular lineages, spatial organization, molecular composition, and interactions, as well as interspecies differences, complicate the interpretation of the results and their extrapolation to humans. Moreover, it is important to note that some of the aforementioned studies were performed using different organisms and were confined to a limited developmental timeframe. However, in the approaches in which different time points have been studied, the datasets could not be used to confirm functional heterogeneity, suggesting that this divergence may be due to a varying differentiation state or a reflection of the transcriptional changes rather than the origin of the distinct subpopulations of cells [43,45]. In the adult heart, scRNA-seq analyses after cardiac injury have suggested the presence of diverse clusters of EPDCs during the reparative response. Furthermore, in most of these studies, epicardial cell characterization was based on known markers and was not further scrutinized; thus, novel subpopulations were potentially overlooked [5]. 

Nevertheless, if there is anything evident regarding epicardial heterogeneity and its composition, it is the need for further analyses to distinguish epicardial cells from EPDCs, in which the transcriptional signature is altered. Although scRNA-seq and high-resolution analyses have recently suggested that there is a heterogenic environment within the epicardial layer, thousands of generated data remain unanalyzed, harboring rare cell populations and interactions within the epicardium waiting to be discovered. It is also important to pay special attention to the developmental stage by performing different analyses at multiple time points during embryonic development and in relation to the adult epicardium after cardiac injury. While maintaining a keen awareness of the cellular context, future studies could greatly benefit from delving into the niche wherein epicardial cells reside. As observed in other experimental models, environmental cues can play a critical role in cellular growth and activity [80,81]. In recent years, several studies have focused on the external signals surrounding epicardial cells, demonstrating their influence on the fate and actions of EPDCs during embryonic development and cardiac repair processes [44,82]. An example is the identification of an epicardial-specific niche within the adult mouse heart, comprising *wt1*^+^ cells encapsulated by ECM components like fibronectin (FN), collagen IV, or hyaluronic acid (HA) [72]. 

Thus, studies conducted during embryonic development and cardiac disease can help us to attain a more comprehensive view of the heterogeneity of the epicardium, allowing for the identification of epicardial subpopulations and their roles during heart formation and in the repair process through either cellular contributions or via paracrine signaling. Such findings could shed more light on the optimization of the post-injury response, leading to the identification of mechanisms that contribute to cardiac repair and regeneration.

## Data Availability

Not applicable.
